# The origins, roles and therapies of cancer associated fibroblast in liver cancer

**DOI:** 10.3389/fonc.2023.1151373

**Published:** 2023-03-23

**Authors:** Natasha Zulaziz, San Jiun Chai, Kue Peng Lim

**Affiliations:** Cancer Immunology and Immunotherapy Research Unit, Cancer Research Malaysia, Subang Jaya, Selangor, Malaysia

**Keywords:** cancer-associated fibroblast (CAF), liver cancer, hepatocellular carcinoma (HCC), immunotherapy, immune-modulating

## Abstract

Hepatocellular carcinoma (HCC) is the most common form of liver cancer. It is often preceded by chronic inflammation such as liver fibrosis and cirrhosis. Different cell types are believed to give rise to liver-specific cancer associated fibroblast (CAF), these include resident fibroblast, hepatic stellate cell, liver cancer cell, hepatic sinusoidal endothelial cell and mesenchymal stromal cell. The abundance of fibroblasts has contributed to the cancer progression, immune modulation and treatment resistance in HCC. In this review, we discussed the origins, subtypes and roles of cancer associated fibroblasts in HCC. Their specific roles in shaping the tumor microenvironment, facilitating cancer growth, and modulating different immune cell types to confer a permissive environment for cancer growth. CAF is now an attractive therapeutic target for cancer treatment, however specific therapeutic development in HCC is still lacking. Hence, we have included preclinical and clinical development of CAF-specific interventions for other cancer types in this review. However, most CAF-specific therapies have resulted in disappointing clinical outcomes, likely due to the difficulties in differentiating CAF from normal fibroblast. A thorough understanding of the characteristics and functionalities of CAF is warranted to further improve the therapeutic efficacy of anti-CAF therapies.

## Introduction

Hepatocellular carcinoma (HCC) accounts for 90% of primary liver cancer (1). It is the sixth most commonly diagnosed cancer and the third most common cause of cancer-related deaths (2). Liver is an important immunological site to mount and resolve inflammation (3). Failure to resolve inflammation often leads to prolonged chronic inflammation. The accumulation of fibroblasts in the chronically inflamed liver is one of the main drivers of liver carcinogenesis (4). Cancer associated fibroblast (CAF) is known to promote the proliferation and invasion of cancer cells by secreting various growth factors and cytokines (5). Lately, the role of CAF in modulating immune responses and complementing the therapeutic effect of checkpoint blockade inhibitors has positioned CAF as an important target for immunotherapy. In this review, we discussed the origins, subtypes, and immune-modulating functions of CAF in HCC, to understand how CAF can improve response to immunotherapy. While the data targeting CAF in HCC is limited, different treatment strategies that have been applied to target CAF preclinically and clinically in other cancers were also discussed.

## Origins of CAF in HCC

CAF is generally defined as persistent activated fibroblasts or myofibroblasts that fail to revert to the quiescent phenotype or undergo apoptosis after the wound is healed. CAF can originate from various cell types, including resident fibroblasts within the tumor, hepatic stellate cells (HSC), and HCC cells (6). Evidence suggests that these cell types have the potential to transform into myofibroblasts, driving fibrosis in the liver, and further promoting HCC through an α-integrin regulated deposition of extracellular matrix (7, 8).

Normal fibroblasts are quiescent and can be activated into a contractile fibroblast, that can interact and influence surrounding injured epithelial cells. Such a state of activated fibroblast is termed “myofibroblast”. Portal fibroblasts which reside underneath the bile duct epithelium were shown to differentiate into α-SMA-expressing myofibroblasts that produce extracellular matrix (ECM) in the portal area in the cholestatic liver fibrosis model (9). Activated HSCs are one of the main sources of ECM in the liver, forming scar tissues during injury. Scarring is intended to protect the liver from further damage during the initial injury. However, as the disease progresses, continuous activation of HSC under the influence of TGF-β can transdifferentiate HSC into α-SMA-expressing myofibroblast, further contributing to cirrhosis and fibrosis (10–13). Fate-tracing studies showed that most liver myofibroblasts originated from HSC (12–15). Another possible source of CAF is HCC cells. Under hypoxic conditions, HCC cells can acquire CAF properties by the influence of cytokines and TGF-β which leads to an increase in the expression of FAP and α-SMA (16, 17), generates mesenchymal cells that resemble fibroblasts, and subsequently contributes to chronic tissue fibrosis and cancer progression (18).

Other cell types such as the hepatic sinusoidal endothelial cells (HSEC) and mesenchymal stem cells (MSC) also been reported to contribute to the CAF-like phenotypes in tumor. Genetic tracing using single-cell transcriptomic analysis comparing fibroblast populations from both normal livers and cancer tissues revealed the presence of endothelial cell signatures in one of the liver cancer fibroblast clusters. Subsequent experiments using *in vitro* model suggest the possibility of HSEC transformation into CAF-like phenotype through an endothelial-mesenchymal transition process (19).

MSCs exhibit different characteristics such as suppressing and promoting tumors during the progression of HCC, therefore, their participation in HCC is controversial. It is believed that MSCs could have tumor suppressing effects in the initial stage of disease by reducing DNA damages, but in the later stage of HCC, MSC played a more tumor-promoting role by enhancing stem cell-like properties and promoting EMT (20, 21). Chemotaxis signals secreted in fibrotic liver or tumor induce the homing properties of MSCs not only from adjacent tissues within the liver, but also from distant sites such as bone marrow (21). The “homing” ability of MSCs to injured tissue further initiate the malignant transformation of HCC (22). Similarly, it is shown that secretion factors from prostate cancer cells induced transdifferentiation of MSC to exhibit CAF phenotype through TGFβ/Smad signaling pathways *in vitro* (23). Although lack of a direct MSC-CAF relationship study in HCC, but differentiation of MSC into fibroblasts and myofibroblasts in the liver, which exhibit functions of repairing injured liver tissues has been demonstrated using an animal model (24). It is possible that MSC could be differentiated into distinct subpopulations of CAF within the HCC tumor especially as a result of chronic liver diseases due to viral infection, NASH or alcoholic liver.

In summary, current evidence has shown that resident fibroblast and HSC are likely the origin of most CAFs in the liver, despite other cell types such as HCC cells, HSEC and MSC within the tumor could also be potential precursors of CAF in HCC. The uncertainty of the origin is likely due to its heterogeneous nature and the origin might differ across different species and animal models. In addition, the lack of specific CAF markers for fated-tracing studies and the lack of longitudinal follow-up on human liver cancer progression also contributed to the ambiguity of CAF origins (25, 26). High-throughput transcriptomic and bioinformatics analysis might shed more light on the origins and formation of CAF in HCC based on their molecular signatures.

## Subtypes of CAF

With the availability of data from high throughput sequencing data, several studies unveiled molecular subtypes of tumor, including CAF. CAF can also be classified into various subtypes based on their phenotypes and molecular signatures. A study that compared the CAF transcriptomic profile of multiple cancer types, including melanoma, head and neck and lung cancer has identified 6 pan-CAF gene signatures: normal, activated, ECM-enriched, pro-inflammation, inflammation with enriched NFκB signaling and cellular proliferation-enriched fibroblasts (27).

Among the cancer types, CAF subtypes in pancreatic ductal adenocarcinoma (PDAC) have been extensively studied. PDAC’s CAF was initially defined phenotypically into 2 subtypes - inflammatory CAF (iCAF) and myofibroblastic CAF (myCAF) (28). Subsequently, another transcriptomic study on PDAC further classified CAFs into 4 subtypes (aggressive, myogenic, immune-enriched, and miscellaneous subtypes) based on their molecular profile and patients’ prognosis (29). In addition to PDAC, molecular profiling of breast cancer CAF also defined CAF into 4 subtypes with different spatial localities. Importantly, the S1 breast CAF subtype is known to modulate immunosuppression by attracting and retaining FoxP3+ regulatory T cells (Tregs) (30).

Similar to the PDAC, fibrosis is also one of the common features of HCC. A study by Galbo et al. found that HCC showed a low to moderate correlation with prognosis after applying the pan-CAF signatures (27). While in other liver malignancies, a single cell-RNA sequencing study on intrahepatic cholangiocarcinoma uncovered two dominant CAF populations, the HSC-derived inflammatory/growth factor-enriched and myofibroblast-enriched subtypes, where these CAF subtypes interact with tumor *via* different mediators and ligand-receptor interactions (31).

These subtypes have different phenotypes, spatial localities, and distribution in the tumor. Different CAF subtypes also displayed different degrees of interchangeable properties of immunosuppression, influenced by different stimuli that were present in the tumor microenvironment. The presence of immune-enriched and non-immune-enriched CAF subtypes is detected in different cancer types, suggesting the importance of CAF in modulating immune responses. Although lack of a system to further classify HCC CAF molecularly, a study using an HSC-selected knockout mice model showed that HGF and hyaluronan-secreting CAFs promote tumor growth while type I collagen-producing CAF plays the opposite role; hinting the presence of different CAF populations in HCC (15). Understanding the subtypes of HCC CAF and identifying markers for each subtype is crucial, which could pave the way for the development of effective therapies targeting the malignant subtypes of CAF.

## CAF promoting tumorigenesis

The abundance of CAF in liver TME has made HCC a fibrotic disease and provides a favorable environment for tumorigenesis.

**CAF promotes cancer cell stemness and self-renewal:** Stem-like cancer cells are cells that acquired the ability to metastasize and form tumor in secondary sites, hence increasing the aggressiveness of a tumor. CAF plays a dynamic role in mediating cancer cell stemness through various signaling pathways. HCC ‘s CAF enhances the stemness through the secretion of paracrine factors such as hepatocyte growth factor (HGF), IL6 and lysine-specific demethylase 1 (LSD1) (32–35). CAF-derived cardiotrophin-like cytokine factor 1 (CLCF1) was also reported to increase the secretion of CXCL6 and TGF-β secretion in an autocrine manner to promote stemness and self-renewal of HCC (36). Elevation and activation of cancer stem-like self-renewal genes such as LSD1 and NOTCH3 were shown strongly associated with poor survival among HCC patients (35).

**CAF promotes vascularization:** HCC is known as a highly vascularized tumor. Unlike vascularization in the healthy liver which has only 20-25% of arterial components, HCC is almost exclusively arterially vascularized (37). CAF promotes vascularization in the tumor by producing various growth factors, chemokines, and ECM to facilitate the recruitment of endothelial cells to form new blood vessels (38). In the human umbilical vein endothelial cells (HUVECs) model, the proliferation, migration, and invasion rate of cells were significantly higher when cultured with CAF isolated from HCC, as compared to non-cancerous fibroblasts (39). CAF also secretes vascular endothelial growth factor (VEGF) and increases the secretion of zeste homolog 2 (EZH2) enhancer that is known to inhibit multiple tumor suppressor genes, including vasohibin 1 (VASH1). VASH1 acts as negative feedback for VEGF expression by inhibiting endothelial proliferation, migration, and invasion. Both VEGF and EZH2 silencing lead to the increased expression of VASH1 and subsequently inhibit HUVECs proliferation and angiogenesis in HCC (39). In another study where transcriptomic analysis of HCC revealed that CD90 expressed by CAF can promote the expression of an embryogenesis growth factor – the placental growth factor (PGF) that promotes neoangiogenesis, a process that allows the tumor to create new blood vessels from pre-existing vasculature in the early stage of cancer development (40).

Besides promoting vessel formation made up of endothelial cells, CAF is also involved in the formation of vascular mimicry (VM) which is shown to be correlated with poor prognosis in HCC (41). Unlike the blood vessel formation process by neovascularization through endothelial sprouting, VM is an alternative form of microcirculation where the malignant tumor cells imitate endothelial cells to form channels for fluid and nutrient transport to the metastasized tumor (42, 43). As the lining of channels is made of tumor cells, the tumor cells are exposed directly to the blood vessels and significantly increase the chances of metastasis. To modulate the formation of VM in liver TME, CAF releases TGF-β, SDF-1, and HGF that bind to their membrane receptors on the tumor, such as TGFβR1, CXCR4 and c-Met, to modify the tumor cell plasticity. The binding of TGF-β and SDF-1 to the membrane receptors on the tumor triggers the expression of endothelial markers like VE-cadherin, matrix metalloproteinase (MMP2) and laminin subunit gamma-2 (LAMC2) that could degrade the ECM to form VM (44).

**CAF enhances ECM remodeling and HCC migration:** ECM in normal cells is important to maintain the architecture and integrity of tissues by forming physical scaffolds. During injury, these physical scaffolds are disrupted, and cross-linking of collagens and elastins in the ECM are activated to regulate the stiffness and integrity of tissues (45). When the damage is resolved in a healthy liver, the stiffness of ECM is reduced by collagen degradation through increased activities of different MMPs. The ECM will then be reverted to a quiescent state.

In the tumor, CAF sculpts the tumor microenvironment through cycles of ECM depository, modification and degradation (46). As HCC lesions often arise from chronic inflammation due to viral or other habits-related factors, deregulation of wound healing and aberrant ECM remodeling are commonly seen due to the persistent activation of CAF. This subsequently increases the aberrant fibrinogenesis, matrix cross-linking and matrix stiffness (47, 48), which further enhances fibrosis and cirrhosis, contributing to HCC development. Matrix stiffness enhances the proliferative capability of HCC cells, therefore also served as a predictor of HCC progression and outcome (49). HCC cells will then activate more myofibroblasts/CAFs and resulted in an accumulation of more ECM proteins in the tumor microenvironment, forming the feed-forward loop of contractility. Increased matrix stiffness also promotes chemoresistance ability, and is responsible for mediating pre-metastatic niche formation in HCC (50).

On the other hand, CAF is shown to be able to secrete matrix-degrading proteases to remodel ECM and pave the ways to facilitate invasion. Through degradation of ECM by secretion of MMPs by CAF, more pro-tumorigenic proteins including matrix-bound angiogenic growth factors are being released, creating a self-sustained tumor microenvironment that is favorable for migration and invasion of tumor cells (46).

**CAF as treatment barrier:** CAF can act as a physical and biochemical barrier, shielding the tumor and inhibiting the exposure of tumor cells to anticancer drugs. CAF causes accumulation of desmoplastic matrix in the TME, such as deposition of laminin A1 and types IV collagen, hindering the delivery of treatment drugs (51). To maintain and support the survival of cancer cells, CAF secretes various secreted factors, creating a tumor-permissive microenvironment. Secreted factors such as the CXC chemokines, TGF-β, IGF, epidermal growth factors (EGF), fibroblast growth factors (FGF), HGF, IL6, IL8, IL10 and IL11 have been reported to support the creation of a drug-resistance tumor microenvironment (52–55). In a NASH liver model, HGF-derived CAF was reported to cause chemoresistance toward cisplatin through activation of the c-MET/ERK/FRA1/HEY1 cascade (34, 56). HGF secreted by CAF binds to receptor tyrosine kinases to further activate the MET pathway, enhancing the crosstalk of cancer cells and hepatic stellate cells (57), resulting in cancer cells’ resistance to chemotherapy (56). Another study published recently dissected the interaction of CAF and liver cancer using 3D co-transplanting of both CAF and liver tumor organoids of mouse and human origin using xenograft models, and treated the tumors with common drugs used in the treatment of HCC. Using either the conditioned medium or the CAF in the culture, the authors managed to demonstrate that soluble factors secreted by CAF contribute to the resistance toward sorafenib, regorafenib and 5-fluorouracil (58).

In addition, CAF is resistant to radiotherapy (59). Upon radiotherapy, CAF do not undergo apoptosis but remained viable to support the recovery of tumor cells. In an *in vitro* model of NSCLC, although radiotherapy impeded the proliferation, migrative, and invasive capacity of CAF (60), CAF can survive ionizing radiation and continue supporting cancer cells recovery by inducing autophagy through the production of insulin-like growth factors-1/2 (IGF1/2) and CXCL12, subsequently promoting tumor recurrence (61, 62).

**Figure 1:**
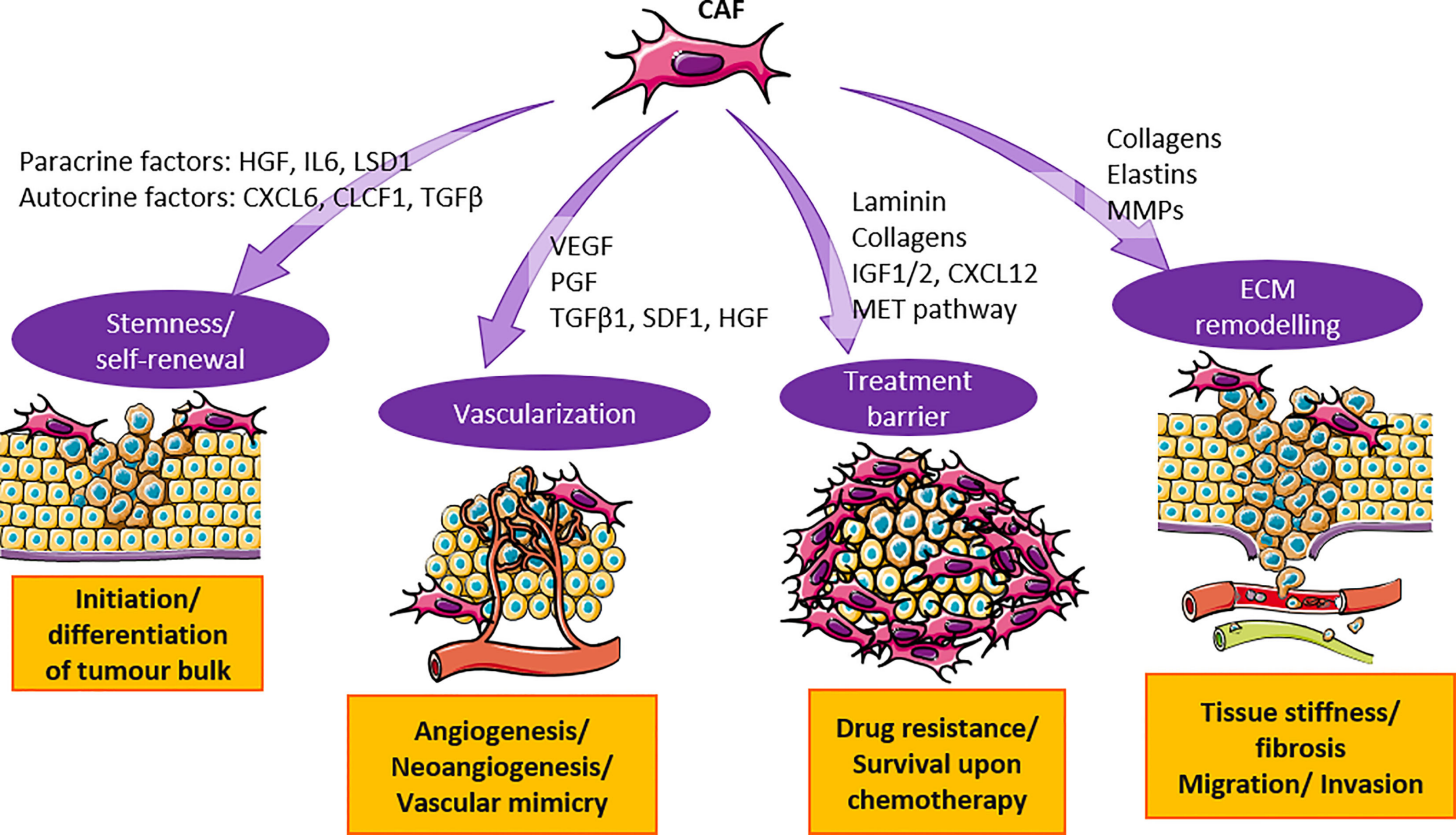
CAF contributes to the tumorigenesis in HCC through secretion of cytokines and activation of signaling pathways. CAF secretes cytokines such as HGF, IL6, TGF-β and others to promote cancer cell stemness and vascularization. They also activate MET and ECM pathway to substantiate treatment barrier and ECM remodeling. Orchestration of events mentioned above promote HCC tumorigenesis.

In summary, CAF has made the TME of HCC a fertile soil that favors cancer growth. CAF secretes cytokines, growth factors, and soluble factors to activate various signaling pathways that contribute to HCC tumorigenesis (**Figure 1**), and promotes cell stemness to initiate the growth of HCC cells. Once the tumor is established, CAF induces vascularization to provide nutrients for the tumor bulk and facilitates the migration of tumor cells to distant sites. The presence of CAF also provides a protective shield for HCC cells against immunosurveillance and the penetrance of treatment drugs.

## CAF restraining tumorigenesis

Although most CAFs exhibit tumor promoting properties, studies in PDAC also pointed toward the other side of the coin for CAFs, suggesting tumor-restraining properties of CAF. It was first discovered in PDAC that depletion of α-SMA+ myofibroblasts in transgenic mice, resulted in the formation of tumors with enhanced hypoxia, EMT and stemness (63). Accumulation of immune-suppressive FoxP3+ Tregs was also observed in the αSMA-depleted animals (63). However, whether such CAF populations are normal fibroblast that has not fully transformed into CAF, is not fully understood. It is hypothesized that during the early stage of cancer progression, myofibroblasts or myCAF have tumor-restraining properties and served as a host defense mechanism (64, 65). The *in vivo* animal model of PDAC showed that inhibition of LIF signaling in iCAFs using JAK inhibitors can shift iCAF phenotypes to populations producing ECM and such changes in the myCAF/iCAF ratio have resulted in tumor control (28). As evidences showed that the CAF subtypes can be interchangeable by different stimuli, reprogramming CAF to quiescent and tumor-restraining myofibroblasts could be a feasible way to target the tumor.

## Immune modulation by CAF

**Figure 2:**
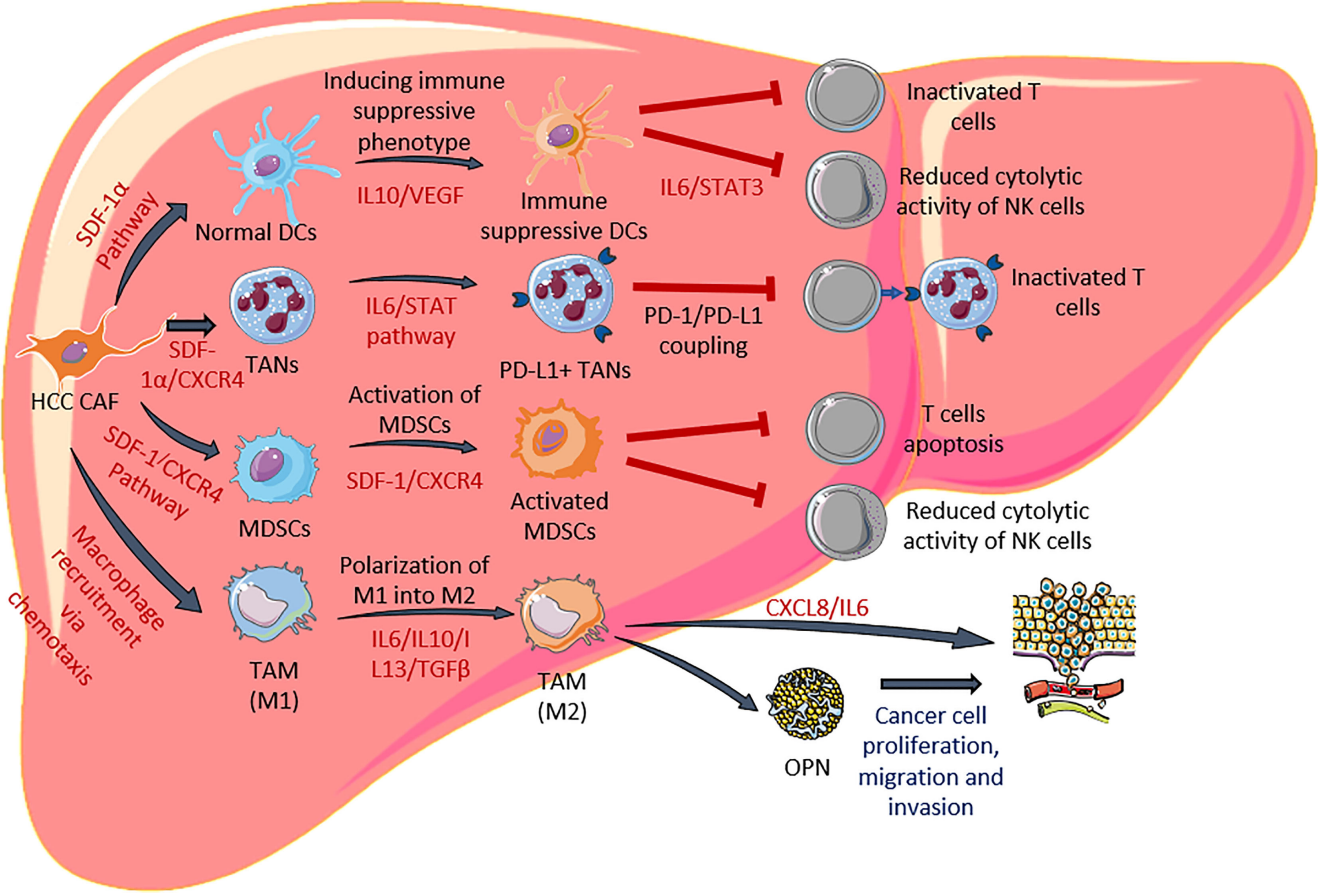
HCC CAF modulates functionalities of dendritic cells, neutrophils, MDSCs and macrophages. CAF secretes various factors to induce differentiation of immune suppressive DCs and PD-L1+ TANs. They are also involved in activation of MDSC and polarization of M2 macrophages. The cascade of these events leads to reduction of effector T cells and NK cells.

In addition to directly promoting the tumorigenesis of HCC, CAF is known to modulate important immune cell types such as dendritic cells and neutrophils to further enhanced cancer progression. Under normal conditions, these immune cells play a pivotal role in the anti-tumor response, however, CAF is capable to change their phenotype to become pro-tumorigenic. Several CAF-induced immune suppressive elements persist within the HCC microenvironment that could lead to impaired anti-tumor response including, dendritic cells (DCs) (66), tumor-associated neutrophils (TANs) (67), myeloid-derived suppressor cells (MDSCs) (68) and tumor-associated macrophages (TAMs). A summary of how CAF modulates the immune response through modification of the abovementioned cells is depicted in **Figure 2** and explained in the following section.

**HCC CAF modulates antigen presentation of dendritic cell (DC):** Dendritic cells play an important role in the activation of naive T cells and initiating an immune response against infection and tumors (69). The main mechanism where DCs promote tumor progression is through dysfunctional antigen presentation (70, 71). Certain cytokines such as IL10 and VEGF secreted by both CAFs and cancer cells are capable to induce immature differentiation of DCs. These immature DCs aid in immune escape by activating Tregs, thus suppressing the function of other effector T cells (72). Immature DCs represent a small subset of regulatory DCs (CD14+ CTLA4+ DC cells) with a high ability for immunological tolerance support and a poor capacity to induce T-cell proliferation (72). These cells express high levels of immunoregulatory cytokines and induce Treg differentiation, thus helping tumor cells dodge immune defenses (66). In addition, this small subset of DCs also expresses inhibitory molecules such as CTLA-4 and PD-1 (72).

HCC CAF plays a pivotal role in manipulating DC phenotype and they have been shown in a mouse model to recruit DCs from the peripheral blood through the SDF-1α-dependent mechanism (73). This CAF-DC has relatively different morphology, characteristics and functions compared to normal DC where they express lower functional markers, such as the costimulatory molecules HLA-DR, CD80, and CD86, mature molecule CD83, and the antigen-presenting molecule CD1a. At the same time, CAF-DCs tend to express more immunosuppressive cytokines, such as IL10, TGF-β and HGF, and fewer IL12p70 and TNF-β in contrast to the normal DCs (73). In addition to that, CAF-DCs are also known to dampen T-cell responses by secretion of IDO through IL6-mediated STAT3 activation and subsequently lead to natural killer (NK) cell dysfunction, characterized by diminished cytotoxicity, decreased cytokine production, low expression of cytotoxic agents and cell activation surface indicators (70, 74, 75). Given the detrimental effects that HCC CAF has on surrounding immune cells such as DCs, strategies targeting CAFs may be beneficial for advanced HCC patients.

**HCC CAF recruits myeloid-derived suppressor cells (MDSC):** The bone marrow produces a diverse population of immature myeloid cells known as MDSC, which are crucial in the suppression of antitumor immunity (76, 77). HCC’s CAF can further amplify this effect by recruiting more MDSC into the tumor site. By stimulating the IL6-mediated STAT3 pathway, CAF can draw monocytes *via* the CXCR4 pathway, which is a receptor for SDF-1, and promote their differentiation into MDSC (68). In HCC, MDSC exerts powerful immunosuppressive effects by enhancing immune checkpoint signaling and dampening NK cell cytotoxicity (78). MDSC expresses galectin-9, a ligand for T-cell immunoglobulin and mucin domain 3 (TIM3), and induces apoptosis upon binding to TIM3 (79). In advanced HCC patients, MDSC can induce PD-L1 expression by interacting with Kupffer cells and inhibiting autologous NK cell cytotoxicity (80).

**HCC CAF recruits tumor associated neutrophils (TAN):** Neutrophils are another type of immune cell that is modulated by CAF and these cells are prominent components of solid tumors (67). Interest in the involvement of TAN in the pathological development of HCC has increased recently. Clinically, the neutrophil-lymphocyte ratio is an independent predictor of survival following hepatectomy in patients with HCC, and TAN plays a significant role in promoting the progression of HCC (81, 82). Furthermore, recent studies suggested that TAN overproduced some chemokines, such as CCL2 and CCL17, which then contributed to HCC progression, metastasis, and resistance to sorafenib treatment. These studies also suggested that TAN mediates the intratumoral infiltration of TAM and Tregs (83). A recently discovered positive feedback loop suggests that TAN upregulates miR-301b-3p expression in cancer cells, sustains hyperactivity in NFkB signaling, and leads to increased levels of C-X-C motif chemokine 5 (CXCL5) secretion, and in turn recruit more TAN (84).

HCC CAF modulates neutrophil functions in several ways. Cytokines secreted by CAF are the main cause of phenotype and function change in neutrophils (85). TGF-β secreted by CAF causes N1 neutrophils to polarize into N2 cells, and the polarization of neutrophils will promote cancer progression as N2 cells stimulate immunosuppression (85). Additionally, CAF also contributes to immune suppression by inducing the differentiation of N1 neutrophils into PD-L1+ neutrophils, which can inhibit T-cell immunity through the IL6-STAT3-PDL1 signaling cascade (67). The same group proposed that CAF modulates neutrophil activity through several steps. First, CAF secretes SDF-1α and draws neutrophils into the HCC. Second, CAF secretes IL6 and activates neutrophils. Through the IL6-STAT3 signaling pathway, PD-L1 expression is also increased in these cells. Third, these activated PD-L1+ neutrophils have a pro-tumor effect by inhibiting T-cell immunity in a PD1/PD-L1-dependent way (85). Currently, atezolizumab/bevacizumab has been approved as one of the first-line regimens for the treatment of unresectable HCC. Atezolizumab works by targeting the PD-L1 check-point inhibitor protein and Bevacizumab works by reversing the effects of VEGF-mediated immune suppression. Although this drug combination seems to be working on advanced HCC patients, however immune suppression exerted by CAF persists and will continue to induce phenotypic changes in these cells.

**HCC CAF recruits and induces polarization of tumor-associated macrophages (TAM):** Similar to neutrophils, macrophages are also modified by HCC CAF through the polarization process. CAF triggers the accumulation of monocytes *via* chemotaxis and drives these monocytes to migrate into the tumor cells. Subsequently, CAF secretes IL6, IL10, IL13, and TGF-β to induce the differentiation of monocytes and M1 macrophages into M2 macrophages (86). M2 macrophages are immunosuppressive where they promote tissue repair and wound healing and it was shown in an animal model infiltration of M2 macrophages leads to multiple features related to poor prognosis in HCC. Once M2 macrophages are activated by CAF, they secrete cytokines like CXCL8 and IL6 that promote HCC metastasis (87). M2 macrophages also release pro-angiogenesis factors such as IL23, PDGF and MMP2 that could further promote tumor progression. More importantly, M2 macrophages recruit Tregs into the tumor, thereby inhibiting effector T-cell activation and proliferation. One unique immune suppression mechanism is that both TAMs and TAM-induced CAFs also secrete osteopontin (OPN) which leads to HCC tumorigenesis (88). OPN is reported to regulate cell-matrix interactions, orchestra cytokine production and mediate cell migration, and administration of osteopontin antibody could significantly suppress the proliferation and migration of HCC (89).

Not only HCC CAF could recruit M2 macrophages to the liver TME, but reciprocally, M2 macrophages could also lead to higher CAF production *via* a positive feedback loop (88). CAF-TAM interaction results in increased EMT in HCC. Both CAF and TAM could secrete plasminogen activator inhibitor-1 (PAI-1), with the aid of CXCL12 secretion by CAFs. PAI-1 is a serine protease inhibitor and high PAI-1 secretion could result in a reduction of E-cadherin and increased vimentin production that drive EMT, and ultimately metastasis (90). Recently, single cell spatial analysis revealed the intricate relationship between FAP+ fibroblasts and SPP1+ macrophages in colorectal cancer patients. They reported that FAP+ fibroblast promote differentiation of a novel subtype of macrophage that consists of both M1 and M2 macrophages and termed it SPP1+ macrophages. This novel subtype will promote the expression of ECM-related genes in FAP^+^ fibroblasts (91). This shows that both FAP+ fibroblasts and SPP1+ macrophages are largely responsible for orchestrating the TME and they should be considered as a potential target for colorectal cancer. Although it is a very interesting finding, such information on HCC is yet to be available.

In summary, CAF induces polarization of M2-TAM that promotes metastasis, angiogenesis, and reduces immune responses in HCC (92). CAFs are densely surrounded by TAMs and the CAF-TAM interactions lead to poor prognosis in HCC. As the tumor, CAFs and TAMs are mutually related and can affect each other *via* a positive loop, the tumor-CAF-TAM relationship could be further explored as a novel therapeutic target for HCC treatment.

## Targeting CAF for cancer therapy

Cancer promoting function of CAF, including its tumor promoting and immune-modulating functions, has made it a promising target for cancer therapy. Moreover, the fibroblast is known to be genetically stable when compared to the epithelial, minimizing the chances of inducing treatment induced resistance. In the following sections, we will discuss different anti-CAF techniques that are being evaluated preclinically and clinically, especially those that targeting (i) CAF specific proteins (FAP, α-SMA and LRRC15); (ii) CAF normalization and (iii) CAF signal.

### Targeting CAF specific proteins

CAFs do not have a specific marker that can delineate them from normal fibroblast or activated fibroblast resulting from the wound healing process. To date, three different markers are known to be expressed by activated fibroblast, these are fibroblast activation protein (FAP), alpha-smooth muscle actin (α-SMA) and leucine rich repeat containing 15 (LRRC15). However, as these proteins are not only expressed in fibroblasts that resided in the tumor, special attention is needed for the management of side effects when targeting cells expressing these three proteins. In the subsequent section, we will discuss different strategies for targeting these proteins with a specific focus on FAP, which has the longest history in the development of CAF targeted therapies.

FAP is a cell surface serine protease expressed by reactive fibroblast especially cancer associated fibroblast (93). Targeting FAP is one of the favorite anti-CAF therapies. Various techniques have been employed either as monotherapy or combination therapy (**Table 1**).

**Table 1:** Available anti-CAF therapies through targeting fibroblast activating protein.

Targeting CAF through FAP				
(A) Preclinical studies				
Approach	Name	Combination	Outcome	Ref
DNA vaccine	–	–	Induced CD8 T cells, suppressed tumor growth in breast and colon murine models	(94)
	Modified synthetic consensus FAP	PSMA, TERT DNA vaccine	Tumor control in lung and breast murine model	(95)
	FAP	Cyclophosphamide	Marked reduced in tumor volume and improved survival in murine breast cancer model	(96)
	Adc68-mFAP	gDMelapoly	Reduced MDSC, TAM, Treg and improved survival in murine melanoma model.	(97)
Small molecule inhibitor or knockout	–	Radiation	Induces T-cell infiltration but not tumor clearance in pancreatic murine model	(98)
Chimeric antigen receptor (CAR)	–	EphA2	Better tumor control and survival in murine lung cancer model	(99)
		CD3z and 4-1BB	Good tumor control in mesothelial and lung murine model	(100)
Recombinant protein	FAP specific scFV fused to PE38 (endotoxin)	Paclitaxel	FAP-PE38 monotherapy resulted in tumor growth; combination with paclitaxel showed significant improvement in tumor control and survival in murine breast cancer model	(101)
	FAP-specific scFV fused to PE38 (endotoxin)	Cancer vaccine targeting gp100, TRP1 and TRP2	Increased CD8 T cells and good tumor control in murine melanoma model	(102)
Viral vector based vaccine	VacV- FAP	VacV-VEGF	Better tumor control in murine prostate cancer model	(103)
	EnAd-FAP-BiTE	CD3e	T-cell activation and fibroblast death	(104)
	ICO15K-FBiTE	CD3	Fibrosarcoma and lung murine model	(105)
Bispecific Antibody	FAP-DR5 BsAb (RG7386)	Irinotecan or doxorubicin	Tumor regression in patient derived xenograft model	(106)
Antibody (Cytokine)	Simlukafusp Alfa (FAP-IL2v)	Anti-PDL1 antibody and CD40 agonist	Better survival in murine pancreatic model.	(107)
(B) Clinical studies				
Approach	Name	Combination	Outcome	Ref
Chimeric antigen receptor (CAR)	FAP-CART		Malignant pleural mesothelial, well-tolerated and CART detected in the periphery	(108)
	FAP-CART	Nectin 4	Not data yet	NCT03932565
Antibody	Sibrotozumab	–	Limited efficacy in metastatic colon cancer, did not meet the primary objective.	(109)
Antibody (Cytokine)	Simlukafusp Alfa (FAP-IL2)	Anti-PDL1 antibody	Tested in advanced and metastatic tumors.	NCT03386721 (110)

Summary of preclinical and clinical studies targeting FAP using different approaches.

**Recombinant protein:** The αFAP-PE38 recombinant protein was generated by cloning the sequence encoding the truncated Pseudomonas exotoxin A (PE38) to the FAP-specific scFv. Monotherapy of αFAP-PE38 on a 4T1 implanted murine model resulted in a reduction in tumor growth *in vivo* and also enhancement of the expression of several growth factors including TGF-β, SDF-1 and VEGF (101). This tumor control efficacy is further enhanced when αFAP-PE38 is used in combination with paclitaxel (101). In addition, the combination of αFAP-PE38 with a cancer vaccine targeting gp100, TRP1 and TRP2 was also tested and resulted in a marked increase in the CD8 T cells and significant improvement in tumor control (102).

**DNA vaccine**: The use of DNA vaccine encoding FAP successfully induced CD8 T-cell mediated killing and significantly suppressed tumor growth in both breast and colon murine models (94). Recently, the use of the FAP DNA vaccine is shown to work synergistically with other tumor antigen-specific vaccine therapies (PSMA and TERT) in lung and breast tumor-bearing mice (95). The combination of DNA vaccine targeting FAP with cyclophosphamide has resulted in an increase in CD8 T cells and a reduction in the Tregs. Importantly, after 3 doses of vaccines and cyclophosphamide, mice inoculated with 4T1 breast cancer cell lines demonstrated impressive tumor control and prolonged survival (96).

**Chimeric antigen receptor (CAR)**: The use of genetically modified T cells expressing FAP-specific CAR was first shown in a murine model. A study by Kakarla et al. demonstrated that combining these FAP-specific T cells with T cells that targeted the EphA2 resulted in enhanced anti-tumor response and improved survival in an A549 tumor model *in vivo* (99). Subsequently, Wang et al. demonstrated the use of CAR construct encoded FAP that coupled to CD3 and 4-1BB domains resulted in good tumor control in both mesothelioma and lung cancer model *in vivo* (100). With these encouraging preclinical data, the use of FAP-specific CAR T cells is now being evaluated in clinical trials.

A phase I clinical trial using FAP-CAR-T-cells on patients with malignant pleural mesothelioma demonstrated that FAP-CAR-T-cell was well tolerated, and persistence of CAR T-cells was detected in the periphery (NCT01722149) (108). In 2019, another Phase 1 clinical trial using an intratumoral injection of Nectin4/FAP-targeted CAR T cells in Nectin4-positive advanced malignant solid tumors has begun patient recruitment, however, no results are released yet (NCT03932565).

**Viral vector-based vaccine**: Some of the FAP-targeting preclinical studies show extensive lethal bone, cachexia and anemia that would caution against the clinical development of systemic FAP-targeted treatments (111, 112). Hence systemic delivery of anti-CAF is not the desired approach as it will mediate toxicity and potentially target normal fibroblasts. The use of oncolytic viruses or virally related vaccine that preferably proliferate in cancer cells is deemed to be plausible as it will promote cytolysis in cancer cells and induces immune cells to kill FAP positive-CAF.

A study by Zhang et al. elegantly demonstrated that depletion of FAP-expressing stroma cells by an adenoviral vector expressing FAP has caused a reduction in the MDSC, TAM and Treg, hence reducing the immune suppressive microenvironment and subsequently enhanced antigen specific responses. B16 murine model that was treated with a combination of AdC68-mFAP and AdC68-gDMelapoly has resulted in complete remission on ~35% of mice and marked improvement in their survival (97). The use of vaccinia virus expressing FAP in combination with vaccinia virus expressing VEGF has improved tumor control efficacy in tumor xenograft model when compared to respective monotherapy (103). Further, the use of oncolytic virus to express a stroma-targeted bispecific T-cell engager (BiTE) that target FAP on CAF and CD3e on T cells (EnAd-FAP-BiTE) in malignant ascites and solid prostate cancer tissues *ex vivo*, has induced T-cell activation and resulted in fibroblast death (104). A similar observation was also reported when oncolytic adenovirus ICOVIR15K was used to generate ICO15K-FBiTE (anti-human CD3 single-chain variable fragment (scFv) linked to an anti-murine and human FAP scFv and assessed in HT1080 and A549 tumor cell lines (105).

**Antibody**: Despite many preclinical studies being conducted to evaluate the efficacy of different anti-CAF therapies, only a handful of drugs entered clinical trials. One of the earliest developments of anti-CAF is the murine monoclonal antibody F19 directed against FAP (113). The humanized version of F19 (Sibrotuzumab) has entered an early phase clinical trial. Of the 17 evaluable metastatic colorectal patients who received at least 8 repeated infusions of Sibrotuzumab, only two showed stable disease and the study did not meet its primary endpoint (109). This questioned the efficacy of anti-FAP as single agent therapy. Hence, the development of bispecific antibodies is now taking over the individual FAP antibody approach and became the mainstream antibody approach for targeting FAP protein. The use of FAP-DR5 bispecific antibody-induced tumor cell apoptosis in a human colorectal xenograft model resulted in marked tumor control when compared to mice treated with only DR5 antibody (106). Noteworthy, there is an additive effect when of FAP-DR5 bispecific antibody is administered together with irinotecan, the current standard of care for colorectal cancer (106).

CAF has also been known for its immune-modulating function. To enhance its efficacy, an antibody against CAF is now combined with a cytokine, IL2 that is known to promote differentiation and survival of CD8 T cells (114). This hybrid antibody is called Simlukafusp Alfa (RO6874281), an immunocytokine comprising of antibody against FAP and an IL-2 variant (IL-2v). Simlukafusp Alfa was shown to activate NK, CD4 and CD8 T cells but not Tregs. Importantly, the use of Simlukafusp Alfa in combination with anti-PD-L1 antibody and agonistic CD40 antibody has resulted in improved survival in a pancreatic murine model. Further, these surviving animals have been protected from tumor rechallenging suggesting that this combination therapy has conferred long-lasting anti-tumor efficacy (107). Simlukafusp Alfa is now being evaluated in combination with an anti-PD-L1 antibody for advanced and/or metastatic solid tumor in a phase 2 basket study (NCT03386721). Data on recurrent and metastatic cervical squamous cell carcinoma demonstrated an overall response rate of 27% with an acceptable safety profile (110).

Another marker of CAF is α-SMA, cells express α-SMA display myofibroblast phenotype which is commonly seen during wound healing, fibrosis and cancer. The tumor promoting role of myofibroblast has made it an attractive therapeutic target. Fibroblast-to-myofibroblast transdifferentiation was reported to depend on a reactive oxygen species generated by NADPH oxidase 4 (NOX4) (115). Inhibition of NOX4 has been shown to reverse the myofibroblast phenotype by reducing the expression of α-SMA. Importantly, the use of anti-NOX4 has successfully controlled tumor growth *in vivo* (115). Contradictory, there are studies demonstrating the use of transgenic mice that selectively target α-SMA positive myofibroblasts resulted in increased tumor invasion and reduced animal survival (63, 116) This is likely due to myofibroblast/CAF that expresses α-SMA plays an important role in secreting extracellular matrix and generating mechanical tension, targeting α-SMA could result in both tumor promoting and inhibiting. Careful selection of therapeutic targets that specifically act upon CAF and not myofibroblast is warranted.

Lately, LRRC15, a protein that is abundantly expressed in cancer with mesenchymal origin (117), is also found to be expressed on TGF-β driven CAF in the lung, head and neck, lung and pancreas (118). Targeting LRRC15 CAF might offer a novel therapy directed against this subpopulation of CAF. The use of antibody drug-conjugate ABBV-085 has successfully prevented metastatic dissemination in ovarian cancer cell lines and patient-derived xenograft models, and showed to reduce cell viability in patient-derived ascites (119). In addition, the use of ABBV-085 has also been shown to inhibit tumor growth in osteosarcoma patient-derived xenografts and soft-tissue sarcomas patient-derived xenografts (120). With these excellent preclinical efficacy data, ABBV-085 was tested in a first-in-human clinical trial and demonstrated to be safe and tolerable. Preliminary efficacy suggested an overall response rate of 20% in patients with osteosarcoma and undifferentiated pleomorphic sarcoma (NCT 02565758) (121, 122).

### Targeting CAF normalization

A study in pancreatic ductal adenoma demonstrated that vitamin D receptor (VDR) is important for the activation of pancreatic stellate cells (precursor of fibroblast), hence targeting VDR is an attractive approach to reduce the activation of fibroblast. Encouragingly, animals treated with vitamin D ligand analog (paricalcitol) have reduced inflammation and fibrosis, subsequently improving the efficacy of gemcitabine in KPC mice (Cre-mediated expression of Trp53R172H and KrasG12D targeted to the pancreas) *in vivo* (123). Following these *in vitro* and *in vivo* findings, a Phase II clinical trial evaluating the use of paricalcitol in combination with gemcitabine and nab-paclitaxel (NCT03520790) or paricalcitol in combination with anti-PD1 is underway (NCT 03331562). Similarly, the use of all-trans retinoic acid (ATRA) has also been shown to induce quiescence in pancreatic cell lines and demonstrated a reduction in Wnt-catenin signaling and cell proliferation when tested in an animal model (124). Importantly, the use of ATRA has been shown to increase CD8 T cells in the juxtatumoral compartment of the treated animals (125). STAR_PAC Phase 1 clinical is initiated to evaluate the combination of ATRA with Gemcitabine and Nab-Paclitaxel in patients with locally advanced or metastatic pancreatic cancer (NCT03307148).

### Targeting CAF signal

The crosstalk between CAF and cancer cells is mediated by different signaling networks including CXCL12-CXCR4 interaction, JAK-STAT3 pathway, TGF-β, IL6, and Hedgehog signaling pathway (126). These specific signals are being targeted to inhibit the function of CAF. CXCL12 contributes to immune cell exclusion and CAFs is one of the sources of CXCL12. Administration of AMD3100, an inhibitor of chemokine receptor 4, a CXCL12 receptor has reversed the CAF-induced immune suppression and synergizes with anti-PD-L1, achieved superior tumor control in a pancreatic tumor model (127). On the other hand, CAF is one of the main sources of TGF-β that facilitates EMT and fibrosis. The use of anti-TGF-β successfully reduces fibrosis in the lung (128). In parallel, the use of an anti-fibrotic agent, Tranilast has been shown to reduce immune suppressive cell types including Tregs and MDSC in an animal model. Promisingly, a combination of Tranilast with a dendritic cell-based vaccine has resulted in better tumor control compared to their corresponding monotherapy in the Lewis lung cancer model (129). Similarly, administration of TGF-β blocking antibody together with anti–PDL1, has successfully increased T-cell infiltration into the tumor bed, and ultimately led to tumor regression in EMT6 and MC38 immune excluded breast tumor models (130).

Another cytokine secreted by CAF is IL6, As IL6 played a major role in promoting cell proliferation, migration, invasion and chemotherapy resistance (52, 131). IL6 is also known to activate STAT3, hence another possible way to target CAF is through inhibiting IL6, IL6 receptor, or JAK. Research on novel agents that target IL6 and its signaling pathway are ongoing preclinically or clinically (132). CAF is also known to induce hedgehog signaling pathway and further promote carcinogenesis. The use of antagonist LDE225 in the PDAC model has demonstrated a reduction in the myofibroblast population, resulting in impaired tumor growth and changes in the immune composition (133). Encouragingly, the use of small molecule inhibitors targeting the hedgehog signaling pathway, saridegib and vismodegib are now being evaluated in early phase clinical trials (NCT01130142 and NCT01195415).

## Concluding remarks

Fibroblast or CAF is an important cell type that carries out multiple functions including ECM deposition and remodeling, promoting angiogenesis, and facilitating signaling pathways of the tumor microenvironment. Targeted therapies against CAF have been developed but resulted in compromised efficacy with extensive toxicities due to the lack of CAF specific biomarker. Lately, the discovery of LRRC15 that uniquely identified TGFβ driven CAF has shed some light on targeting CAF while sparing normal fibroblast. In addition, data on targeting the CAF-regulated microenvironment by inhibiting NOX4 is also promising. There is continuous effort to develop novel approaches that can increased the treatment specificity for example the use of CAR-T therapy, oncolytic viruses or intratumoral injection. Preliminary data from Phase 1 clinical trial using CAR-T-FAP injecting into the pleural cavity of malignant pleural mesothelioma patients have demonstrated the expansion of the antigen-targeting T cells (99, 134). The use of checkpoint blockade inhibitors has become a new standard of care for many cancers. However, the overall response rate is varied and we believe it can be further improved by addressing the deleterious effect of CAF, for example the recruitment of various immune-suppressive molecules including Treg, DC, TAN, MDSC and TAM. Promisingly, the use of Simlukafusp alfa, an immunocytokine in combination with atezolizumab has achieved a disease control rate of 71% in 44 response-evaluable patients (110). Although specific CAF therapy for HCC is still lacking. The desired intervention will need to preserve the underlying physiological function of the liver while destroying liver cancer cells. The discovery of specific markers that can efficaciously delineate CAF from normal fibroblast for example the newly reported LRRC15 might be able to address this gap.

In summary, despite some inherent difficulties in specifically targeting CAF, researchers are trying different approaches to increase the treatment specificity, for example intratumoral anti-CAF delivery that might overcome the undesired toxicities. Many early phase clinical trials are initiated in these two years, we are anticipating for their results.

## Author contributions

NZ, SC, and KL were responsible for the conceptualization, writing, reviewing & editing of the manuscript. NZ and SC contributed to generating figures. KL was responsible for study supervision. All authors contributed to the article and approved the submitted version.
